# Supplementary figures and images for: Viral Diversity in Benthic Abyssal Ecosystems: Ecological and Methodological Considerations
Source: Viruses. 2023 Nov 21;15(12):2282. doi: 10.3390/v15122282 (PMC10747316; doi:10.3390/v15122282)

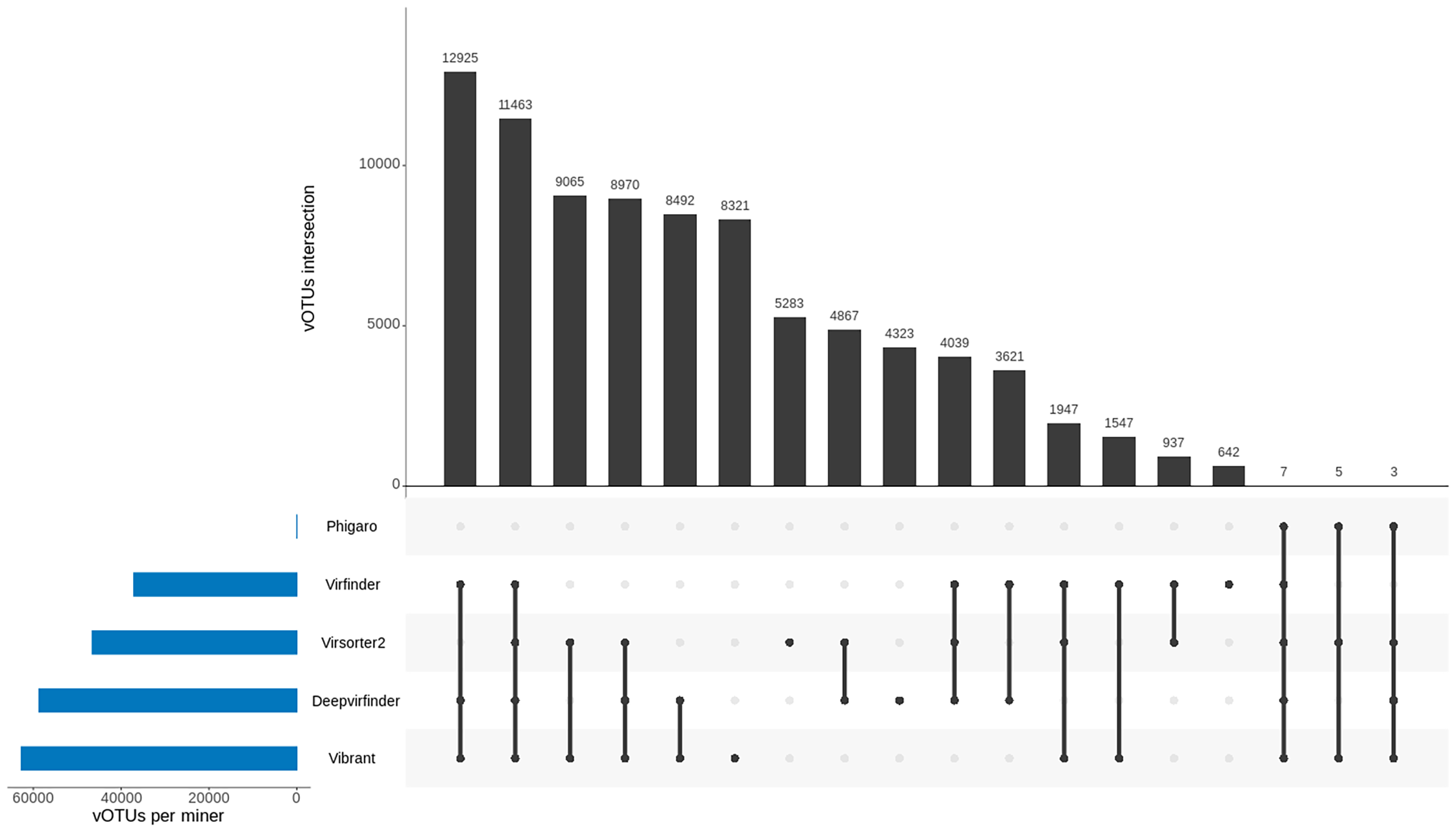

Supplement: Supplementary file 1 [file viruses-15-02282-s001.zip › Figure S1.tif]

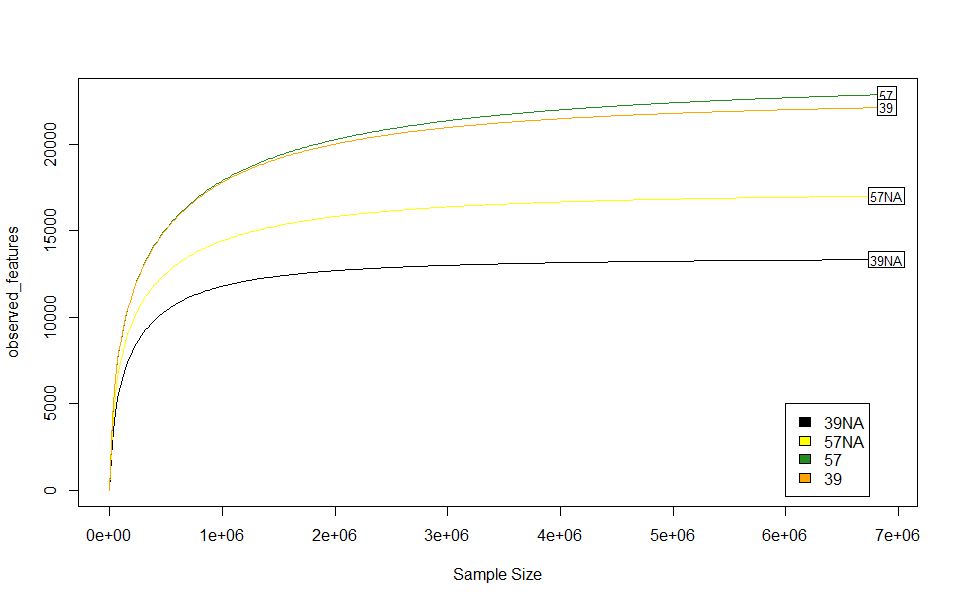

Supplement: Supplementary file 1 [file viruses-15-02282-s001.zip › Figure S2.tiff]

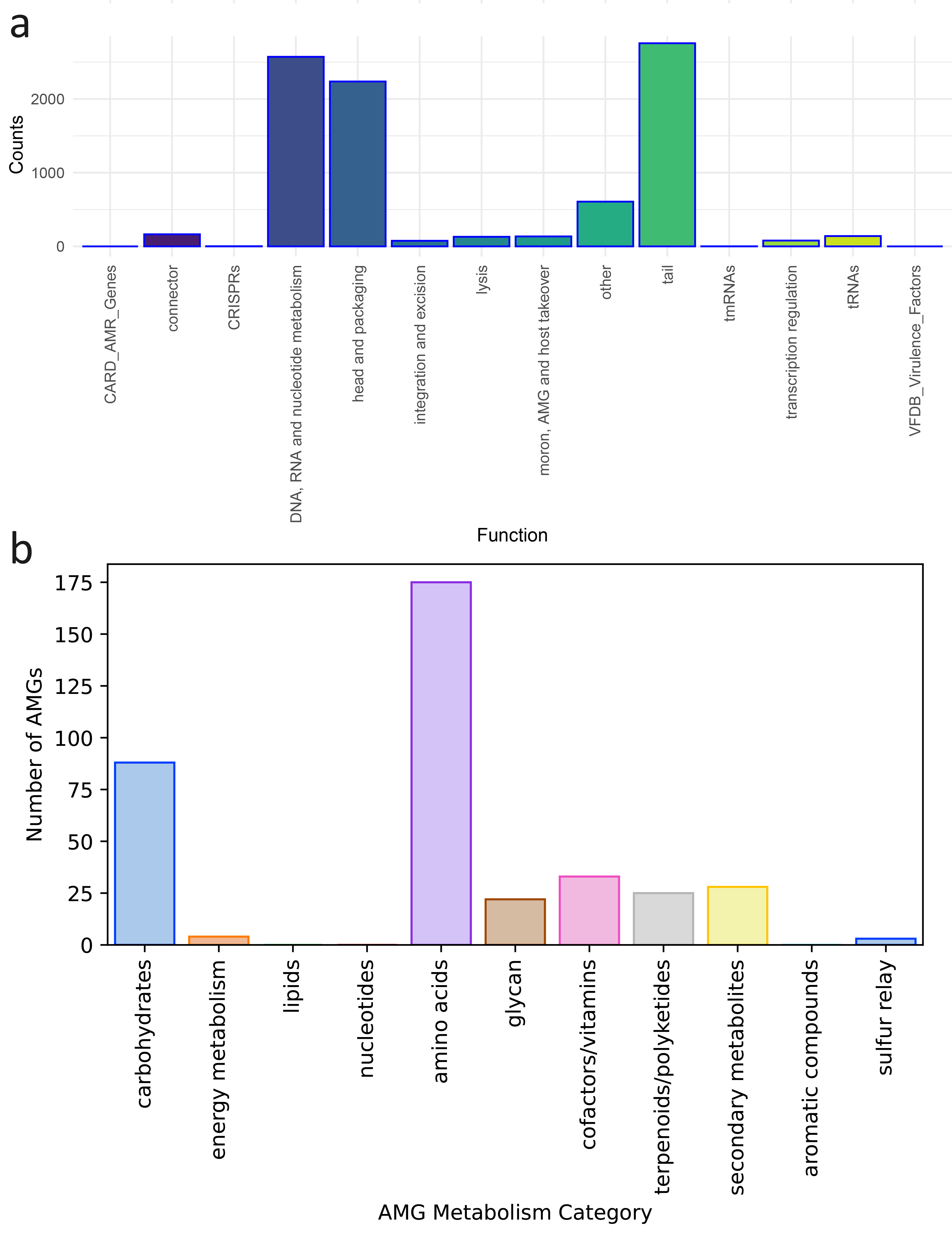

Supplement: Supplementary file 1 [file viruses-15-02282-s001.zip › Figure S3new.tif]

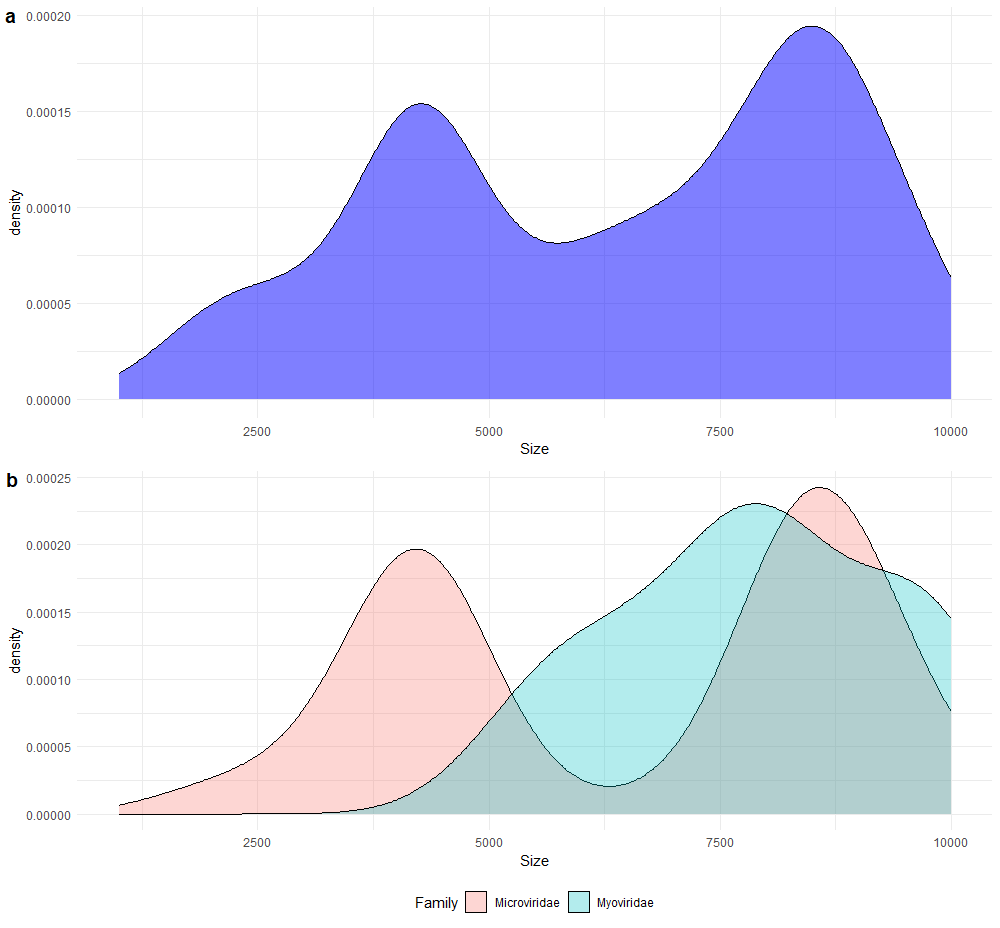

Supplement: Supplementary file 1 [file viruses-15-02282-s001.zip › Figure S4new.tiff]
